# A study on motion reduction for suspended platforms used in gravitational wave detectors

**DOI:** 10.1038/s41598-023-29418-x

**Published:** 2023-02-10

**Authors:** Sina M. Koehlenbeck, Conor M. Mow-Lowry, Gerald Bergmann, Robin Kirchoff, Philip Koch, Gerrit Kühn, Johannes Lehmann, Patrick Oppermann, Janis Wöhler, David S. Wu

**Affiliations:** 1 Max Planck Institute for Gravitational Physics (Albert Einstein Institute), 30167 Hanover, Germany; 2 Institut für Gravitationsphysik, Leibniz Universität Hannover, 30167 Hanover, Germany; 3 Faculty of Science, (Astro)-Particles Physics, Vrije Universiteit Amsterdam, 1081 HV Amsterdam, The Netherlands; 4 Fritz Haber Institute of the Max Planck Society, 14195 Berlin, Germany

**Keywords:** Astronomical instrumentation, Techniques and instrumentation

## Abstract

We report a reduction in motion for suspended seismic-isolation platforms in a gravitational wave detector prototype facility. We sense the distance between two seismic-isolation platforms with a suspension platform interferometer and the angular motion with two optical levers. Feedback control loops reduce the length changes between two platforms separated by $11.65\,\textrm{m}$ to $10\,\mathrm {pm\,Hz}^{-1/2}$ at $100\,\textrm{mHz}$, and the angular motion of each platform is reduced to $1\,\mathrm {nrad\, Hz}^{-1/2}$ at $100\,\textrm{mHz}$. As a result, the length fluctuations in a suspended optical resonator on top of the platforms is reduced by three orders of magnitude. This result is of direct relevance to gravitational wave detectors that use similar suspended optics and seismic isolation platforms.

## Introduction

Today’s interferometric gravitational wave observatories, the Advanced Laser Interferometer Gravitational-Wave Observatory (LIGO)^1^, Advanced Virgo^2^, and Kamioka Gravitational Wave Detector (KAGRA)^3,4^, provide a direct way to study compact astrophysical objects such as Black Holes and Neutron Stars^5^. When two of these objects orbit each other and merge, they emit gravitational waves strong enough to be detected on earth^6^. Laser interferometers at the observatories measure the gravitational wave induced space-time distortion by comparing the length of two orthogonal kilometer-long sections of space^7^.

The natural and anthropogenic motions of the Earth’s surface are larger than the length changes from gravitational waves. The laser interferometer optics are decoupled from the ground motion through a passive suspension system of mechanical filters. The ground motion is attenuated by them, and the residual motions on the measurement paths are small enough for the gravitational wave measurement. The suspension of the interferometer optics provides a free-falling inertial measurement frame^1,2^. The core optics of the laser interferometer are therefore referred to as test masses. The filter response of the suspension system determines the lower frequency limit for scientific observations. The suspension has several stages, each providing isolation inversely proportional to the square of the Fourier frequency, with cut-off frequencies between a few tens of millihertz and a few hertz. The ground motion transmitted to the test masses is strongly attenuated at frequencies above about $10\,\textrm{Hz}$^8,9^. The suspension systems are mounted on an actively controlled ‘seismic isolation system’ that reduces inertial motion near the resonant frequencies of the suspension and allows for large-scale position control. Examples of these platforms are the Horizontal Access Modules Intra-vacuum Seismic Isolators (HAM-ISI) in the LIGO detectors^10^, and the Albert Einstein Institute Seismic Attenuation System (AEI-SAS)^11^ used in this experiment.

Stable operation of the interferometer requires active positioning of the optics^12^. The stability and linearity of the interferometer response place stringent requirements on the frequency response of the feedback filters. The gravitational wave signal is composed of the control signal and the error signal of the differential arm length change of the interferometer. However, noise in the interferometer signal can be introduced by the control of auxiliary length degrees of freedom. The is not completely linear or stationary and an offline subtraction from the interferometer signal leads to a partial improvement^13^. The opto-mechanical interaction of the laser light with the suspended optics further leads to unstable Sidles-Sigg modes^14^, which also need to be actively stabilized.

Excess noise from the feedback control loop reintroduces noise into the interferometer measurements. Improvements to the active control system are regularly implemented, but the observation band of the LIGO interferometers at frequencies below 25 Hz is still contaminated by noise introduced by control loops^13^. This is a persistent problem with the current generation and one of the major challenges for the future observatories Einstein Telescope and Cosmic Explorer^15,16^. Reduced input seismic motion to the mechanical filter chain would be a solution and is a criterion for the location of future observatories^17,18^. However, the ground motion at the detector site cannot be changed, and there are natural limits to it^19^.

We demonstrate how an interferometric and optical position sensor can artificially reduce input motion in an upstream stage of the seismic isolation chain. This approach has been studied before and was called a Suspension Point Interferometer, see Ref.^20^. A gravitational wave detector is sensitive to length changes between its optics. Measuring and compensating the differential suspension point motion will reduce the differential length fluctuations between the suspended test masses. As Ref.^20^ reports, this was first proposed by R.W.P. Drever, and is detailed in Ref.^21^, later a demonstration was reported in Ref.^22^. In existing observatories the ‘suspension points’, the points from which the test masses are passively suspended, are often obstructed. However, we can stabilize the differential motion further upstream by installing the auxiliary interferometer on the seismic isolation system that serves as a platform for mounting passive suspension chains. This concept is called a Suspension Platform Interferometer (SPI), and has been studied in Ref.^23^ and Ref.^24^. The first iteration of the SPI described in Ref.^24^ did not offer sufficient sensitivity for a virtual suspension point stabilization, and the concept has been altered to incorporate optical levers for angular pitch and yaw control of the independent seismic isolation platforms.

In this paper, we demonstrate the feasibility of using a suspension platform interferometer to stabilize the length of a suspended optical resonator. We explain the concept and the experimental results obtained at a gravitational wave detector prototype, the AEI 10 m-Prototype. We report a reduction of longitudinal motion of up to four orders of magnitude for the length between two seismic isolation platforms and improve the rotational stability of the platforms by a factor of 50 and 100 for pitch and yaw respectively. We thereby stabilize the length of a suspended optical resonator with a baseline greater than ten meters and reduce the differential motion by three orders of magnitude. The results are directly relevant for gravitational wave detectors since their optics are distributed across large base lines and the optics are individually suspended.

## Results

The results are split into three parts, the length and angular stability at the level of the seismic isolation platforms, and the resulting length stability of a suspended resonator on top of them. The first two sets of measurements were performed with two isolated platforms, each supported by an AEI-SAS. A detailed description of the AEI-SAS can be found in Ref.^11^ and Ref.^25^, and the experiment is described in the Methods section. We measure the length fluctuations along an $11.65\,\textrm{m}$ baseline between these two seismic isolation platforms as well as the angular stability of each platform in two degrees of freedom: Pitch and Yaw. The AEI-SAS has active feedback control in six degrees of freedom, and we will show that we can improve the isolation performance of the AEI-SAS with SPI optical sensors. The standard operating mode for the AEI-SAS uses geophones, accelerometers, and relative position sensors to ground in closed-loop feedback control^26^. The input-noise reduction for gravitational wave detectors is studied with a suspended optical resonator extending over two AEI-SAS platforms. It has a round-trip length of $21\,\textrm{m}$, with three mirrors suspended from frames about $0.8\,\textrm{m}$ high.

### Length stability at the platform level

**Figure 1:**
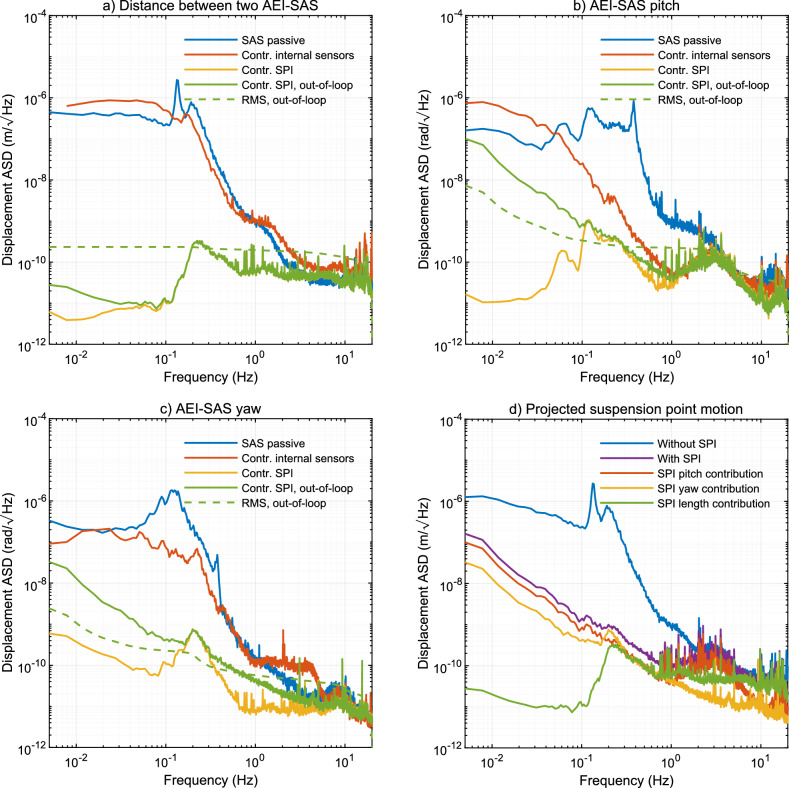
The first three graphics (**a**-**c**) show the residual Amplitude Spectral Density (ASD) of displacement in length and angle of the AEI-SAS platforms. All data was measured with the sensors of the SPI. Graphic (**a**) shows the residual length fluctuations between two platforms, graphic (**b**) shows the pitch of an individual platform, and graphic (**c**) shows the yaw of one platform. The AEI-SAS can be used without active control, and its passive performance is shown in blue. It is equipped with inertial and relative displacement sensors to the ground. The residual displacement with feedback control with the internal sensors and actuators applied is represented by the red lines. The feedback control with the SPI sensor input is represented by the yellow lines. An additional SPI sensor is used for an independent measurement and is shown in green and the Root-Mean-Square of it is shown in the dashed green curve. (**d**) Shows the projection of the suspension point motion for the suspended optical resonator, in the direction of its optical axis, extrapolated from the seismic platform motion. The blue curve shows the expected suspension point motion without a SPI and the purple curve with a SPI. The red, yellow, and green curves show the effects of pitch, yaw, and length of the seismic isolation platforms on the suspension point displacement.

To analyze the benefits of a SPI system, the isolation performance of the AEI-SAS was analyzed in three different distinct control states were used: passive-only, where isolation is provided by its mechanical compliance; internal control, where internal displacement and inertial sensors are used; and SPI control, where the interferometric and angular sensors of the SPI are used. Additional out-of-loop SPI sensors were used to verify performance. Figure 1 shows the results of the AEI-SAS performance data. Figure 1a shows the interferometric measurement of the displacement stability of two AEI-SAS separated by $11.65\,\textrm{m}$. The blue line shows the passive performance of the AEI-SAS. The ground and suspended platform move synchronously below the mechanical resonant frequency of the SAS, approximately $120\,\textrm{mHz}$ for this degree of freedom, and no passive isolation is provided. At the resonant frequency the motion of the ground is amplified and above this frequency the system passively isolates ground motion proportional to $f^{-2}$^25^.

The instrumentation of the AEI-SAS includes six inertial sensors, six relative displacement sensors, and six co-located actuators. The three translational and three rotational degrees of motion can be actively stabilized. The red line shows the displacement stability along the $11.65\,\textrm{m}$ baseline when the two AEI-SAS are individually controlled by their internal sensors. The displacement stability is improved at the resonant frequency. The passive and active performance curves were measured at times with slightly different ground motion, and that is responsible for most differences between the curves away from the resonant frequency. A detailed study of the internal sensors and controls can be found in Ref.^25^ and Ref.^26^.

Instead of stabilizing the individual AEI-SAS with their internal inertial sensors, we can use the interferometer of the SPI to stabilize the differential length change between two of them. We force the remote AEI-SAS to follow the central AEI-SAS in the direction of the laser beam and hence the differential motion is suppressed. See the schematic overview in Fig. 3 for more details. All remaining degrees of freedom are stabilized with the internal sensors.

The in-loop signal of the SPI interferometer is shown as a yellow line in Fig. 1a. When compared to the passive or active stabilization with the internal sensors, we can see that the differential motion is suppressed by more than four orders of magnitude, down to $10\,\mathrm {pm\, Hz}^{-1/2}$ at $100\,\textrm{mHz}$. This is confirmed by an out-of-loop measurement with an independent photodetector in the second output of the interferometer and is shown by the green line.

### Angular stability at the platform level

In order to transfer the length stability along the laser beam at the platform level to the suspended optics, two additional degrees of freedom have to be improved. The optics of the scientific instruments are not rigidly attached to the AEI-SAS platform, instead, they are suspended as cascaded pendulums from large frames to provide additional passive isolation. As a result, the suspension point of the optics is offset from the platform’s center of rotation. As the platform tilts, the suspension point moves along an arc, resulting in a translation of the optic and a change in the length of the science interferometer. The same is true for optics that are not placed on the central axis. When the platform rotates a length change is induced in the interferometer.

Laser beams from the SPI are used as optical levers to measure the pitch and yaw degrees of freedom of the AEI-SAS more accurately than the local inertial sensors. The optical lever is a simple device where a laser beam is launched from one AEI-SAS and detected with a segmented Quadrant Photodetector (QPD) on a second AEI-SAS located $11.65\,\textrm{m}$ away, see Fig. 4. Being separated by more than ten meters, the rotation of the first AEI-SAS becomes the dominant source of displacement of the laser beam on the QPD.

To analyze if the optical lever is an adequate solution to stabilize the pitch and yaw degrees of freedom of the AEI-SAS, we performed an analysis similar to the length analysis above. In Fig. 1b,c respectively, the pitch and yaw displacements for one AEI-SAS are shown. The passive performance of the AEI-SAS is again shown in blue lines, and the active performance when using the internal sensors is shown in red.

The stability of the AEI-SAS platforms are further improved by using the optical lever signals. Replacing the internal sensor signals with the optical lever signals results in a peak reduction of motion of more than two orders of magnitude for the yaw degree of freedom and a factor of up to 50 for the pitch degree of freedom. The improved motion of pitch is $1\,\mathrm {nrad\, Hz}^{-1/2}$ and yaw motion is $0.5\,\mathrm {nrad\, Hz}^{-1/2}$ at $100\,\textrm{mHz}$.

The in- and out-of-loop measurements for pitch and yaw motions show that the control loop gain is not limiting the performance and there is additional noise on the out-of-loop sensor. The electronic noise and readout noise are discussed in the Methods section and are below the out-of-loop optical lever measurement. The excess noise could be due to other motions that cause the laser spot on the photodetector to shift. One example is the displacement of the photodiode orthogonal to the propagation of the laser beam. These effects were investigated, but no clear results could be obtained because the sensor noise for these degrees of freedom were larger than the measured effect.

Figure 1d shows the projected suspension point motion of an optical resonator installed on top of two seismic isolation platforms. With the improved control of the AEI-SAS by the optical lever signals, the length change of the optical resonator is much reduced. However, the residual motion below about $200\,\textrm{mHz}$ is still dominated by the pitch and yaw motion of the AEI-SAS, which in turn translate the mirrors of the optical resonator along its optical axis. In the next section a series of measurements with the optical resonator show how pitch, yaw, and length controls of the SPI affect the resonator length.

### Length stability of a suspended optical resonator

**Figure 2:**
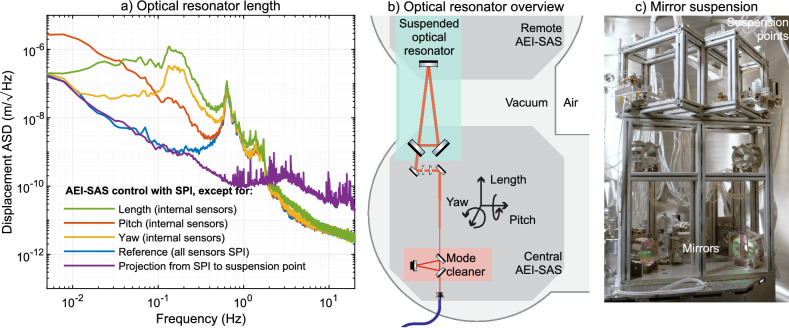
The figure shows a measurement of the length fluctuations of an optical resonator used to test the suspension point stabilization. The purple curve is taken from Fig. 1d and shows the expected length change of the optical resonator at its suspension point with a SPI system and matches the measured length change of the resonator in blue at frequencies below its suspension resonance. The green, red, and yellow curves show the length of the optical resonator without length, pitch, and yaw controls at the seismic platform. It is clear that pitch and yaw control by the SPI is essential for good performance. A simplified schematic shows the setup of the experiment, and the photo shows two of the three suspended mirrors of the optical resonator. The third mirror is located on an AEI-SAS about 10 m away.

To test the reduction of motion in a gravitational wave detector-like system, a suspended optical resonator of the AEI 10 m-prototype was used as a test bed. A simplified schematic overview and a picture can be found in Fig. 2. The triangular suspended optical resonator provides the frequency reference for the main science laser. All changes in length along the path of the resonator are imprinted on the laser frequency. The resonator has a round-trip length of $21\,\textrm{m}$, and extends over a baseline of more than $10\,\textrm{m}$. It has three mirrors individually suspended from frames about $0.8\,\textrm{m}$ high. Two mirrors are grouped on the central AEI-SAS and the third mirror is on the remote AEI-SAS. A second optical resonator is used to measure the laser frequency fluctuations and thus serves as an independent sensor for the changes in the length of the suspended optical resonator. It is constructed with a monolithic block of Super Invar as a spacer of the mirrors, with a round-trip length of $0.5\,\textrm{m}$. It serves as a spatial-mode cleaner for the laser input beam in the AEI 10 m-Prototype. It will be called the ‘mode cleaner’ from here on, to reduce confusion with the suspended optical resonator under test. The laser light is delivered with an optical fiber. Both the optical fiber and the mode cleaner are rigidly attached to the central AEI-SAS, providing common-mode suppression of the AEI-SAS motion.

The SPI performance is evaluated for frequencies below $0.64\,\textrm{Hz}$, which is the fundamental suspension resonance of the mirror suspensions. The optical resonator has a feedback control system for the mirror motion to suppress motion enhancement at the pendulum resonances actively. It was still in commissioning at the time of this experiment and was limited by its inherent noise. Figure 2 shows the residual length variations imprinted on the laser frequency. All measurements were made with the mode cleaner and were calibrated to represent the length changes along the baseline of the optical resonator.

To understand the coupling of the seismic isolation of the AEI-SAS to the suspension points of the optical resonator, the feedback control was switched from SPI to internal sensors. The reference trace is the state of the lowest displacement noise in the optical resonator. It is represented by the blue line in Fig. 2, where the sensor input for the feedback control of the two AEI-SAS in length, pitch, and yaw is provided by the SPI. Each degree of freedom is switched to the internal sensors of the AEI-SAS. The red line represents the state where the length and yaw are controlled with the SPI signals, but the pitch degrees of freedom for both AEI-SAS are controlled by the internal sensors. For the yellow line, the length and pitch are controlled with the SPI signals, and the yaw is controlled with the internal sensors of the AEI-SAS. The green line represents AEI-SAS control, where length is controlled with the internal sensors, and pitch and yaw are controlled with the SPI.

The length stability at the platform level would have been ineffective for the optical resonator without the improved angular stability through the optical lever. When we compare the measurement represented by the blue line to the yellow line, we see how the optical resonator length is dominated by yaw of the two AEI-SAS platforms it is installed on. The offset from the center line of the AEI-SAS rotates the optics along an arc and displaces them along the center line of the resonator. The offset is approximately $0.6\,\textrm{m}$ in the direction along the laser beam and from the center line of the AEI-SAS. When we compare the measurement represented by the blue line to the one represented by the red line, we see how the pitch rotation of the two AEI-SAS couples to the length change in the optical resonator. The coupling is proportional to the height of the suspension point above the AEI-SAS platform and the offset is $780\,\textrm{mm}$.

The angular and length stabilization of the AEI-SAS platforms resulted in a resonator length stabilization of a factor of 1000. The out-of-loop measurement shown in Fig. 1 for length and angle were used to project the suspension point motion when all SPI sensors are used to control the AEI-SAS. It is shown in the purple line. The projected suspension point motion and measured resonator length motion agree below the fundamental suspension resonance of the resonator mirrors.

## Discussion

In this report, we demonstrated a seismic motion reduction in a gravitational wave detector prototype by three orders of magnitude. The input motion into a suspended optical resonator with a baseline of $10\,\textrm{m}$ distributed across two independent seismic isolation platforms was reduced via the stabilization of its suspension points. The interferometer and optical levers of a SPI provided signals for active control of the AEI-SAS seismic isolation system. A stabilization in the platform motion resulted in a subsequent stabilization of the suspension point. The length between two AEI-SAS was controlled to $10\,\mathrm {pm\, Hz}^{-1/2}$ and the angle of the individual platforms to $1\,\mathrm {nrad\, Hz}^{-1/2}$ at $100\,\textrm{mHz}$. The optical resonator length was reduced by three orders of magnitude to $1\,\mathrm {nm\, Hz}^{-1/2}$ at $200\,\textrm{mHz}$.

As described in Ref.^13^, gravitational wave detectors must suppress the length and angle motions of the interferometer optics and optical resonators to reach and hold the interferometer at its operating point. The control loops are optimized to introduce as little noise as possible into the gravitational wave band and yet they are still a dominant source of noise. As discussed in Ref.^27^, a desirable approach to address angular control noise for a gravitational wave detector at 5 Hz is to reduce the tilt of the seismic isolation platform to $100\,\mathrm {prad\, Hz}^{-1/2}$ in the range 10–500 mHz. Our results show that optical levers have the potential to achieve this noise level and can provide a stability at $100\,\textrm{mHz}$ better than $1\,\mathrm {nrad\, Hz}^{-1/2}$. This is more than an order of magnitude better than the GS-13 geophone sensors installed in LIGO’s HAM-ISI seismic isolation systems^10^. As the geophones provide better high frequency sensitivity, optical levers could be used to complement them below $1\,\textrm{Hz}$.

It is also discussed whether SPI stabilization could reduce noise in gravitational wave detectors caused by stray light. As described in Ref.^28^, the large amplitude slow differential motion between seismically isolated optical benches at Advanced Virgo results in an up-conversion of the scattered light signal to the gravitational wave detection band. Thus, seismic noise, e.g., at the micro-seismic peak at about 300 mHz, is converted to frequencies greater than 10 Hz.

Compared to the original results of Ref.^20^, the SPI at the AEI 10 m-Prototype achieves a factor of 10 greater suppression, with slightly improved displacement noise in a suspended optical resonator. We believe that length and angle stabilization at the seismic isolation platform is preferable to stabilizing the ‘suspension point’ within the passive suspension chain of an optic. First, because the optics’ suspension point in the gravitational wave detector is often occluded, and second, because the seismic isolation platforms often carry complex payloads with multiple optics and other instrumentation that will all benefit from reduced differential motion.

The demonstrated feedback control with SPI signals at the seismic isolation is a way to reduce low frequency seismic noise and control noise in the laser interferometers and optical resonators of gravitational wave detectors.

## Methods

**Figure 3:**
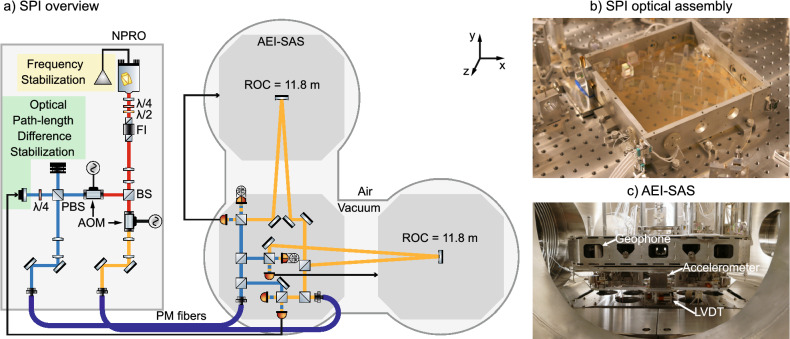
The simplified schematic shows the length sensing of the SPI in the AEI 10 m-prototype. The vacuum system houses the interferometer optics. The light is injected via optical fibers. On the left side of the schematic is the laser preparation for the SPI. In the upper right photograph the SPI hardware can be seen. An aluminum cover protects the central optics during operation. They are assembled on a ULE base plate. The AEI-SAS is shown in the bottom right photograph with some of its internal instrumentation labelled. The SPI is sitting in the center on top of the central AEI-SAS platform. The degrees of freedom are: Length along the y-axis, pitch around the x-axis and yaw around the z-axis. The AEI-SAS in the right half of the schematic overview was not installed at the time of the experiments.

Gravitational wave detectors are complex infrastructures with restricted access for research and development projects. Instead, concept tests are done on bench-scale experiments and feasibility studies are performed on large scale in prototype systems for gravitational wave detectors. The AEI 10 m-Prototype is designed to serve as such a test bed for future gravitational wave detectors^29,30^. The necessary infrastructure is being set up, and one of the first experiments will be a Fabry-Perot Michelson interferometer limited by quantum noise in the form of shot-noise and back-action noise from radiation pressure. Multi-stage isolation of the interferometer optics is necessary to suppress the ground motion to a level below the Standard Quantum Limit (SQL) of interferometry. The first isolation stage is the AEI-SAS, which suspends the optical tables as suspended platforms^25^. A photograph is shown in Fig. 3. Three of these suspended platforms are arranged in an L-shaped configuration, separated by $11.65\,\textrm{m}$, as seen in the schematic overview in the same figure. Core optics are further isolated using multi-stage suspensions.

For low frequencies from $10\,\textrm{mHz}$ to $1\,\textrm{Hz}$ additional isolation along the beam axis of the sub-SQL interferometer is desired to reduce the suspension point motion of the optics and therefore the actuation needed for the interferometer length stabilization. The SPI was developed to provide this additional stability for all experiments with optical components distributed across multiple AEI-SAS^24^.

The SPI is a low-frequency sensor for differential platform motion along the beam axis and it is for feedback control of the AEI-SAS. The angular degrees of freedom are stabilized at the platform with signals generated by optical levers running between the AEI-SAS. The feedback forces are applied the AEI-SAS’s voice-coil actuators that are co-located with the internal displacement sensors.

All data was processed using high-level tools within the LIGO-type Control and Data System (CDS) installation that was adapted for use at the AEI 10 m prototype^31^. The Amplitude Spectral Densities (ASD) are computed either with MATLAB and a graphical user interface called LigoDV or LIGO’s ‘Diagnostic Test Tool’. The data was windowed with Hanning windows.

### Length sensing

The interferometer of the SPI measures the differential length motion between two AEI-SAS. The interferometer layout is shown in Fig. 3 in a simplified schematic. A detailed description of the design can be found at Ref.^24^. The original hardware of the SPI was adapted from experiments for the LISA Pathfinder mission at the AEI. The requirements overlapped, and it could have served as a test bed for LISA pathfinder experiments as well^32^.

In total, the SPI consists of four heterodyne Mach-Zehnder interferometers with a laser wavelength of 1064 nm. In the schematic overview in Fig. 3 only three interferometers are shown, as one is redundant. Laser light is prepared outside of the vacuum system on an optical bench. A Nd:YAG Non-Planar Ring Oscillator (NPRO) (InnoLight P20NE) generates 1 W of laser light, of which approximately $50\,\textrm{mW}$ is used. The frequency shift for the heterodyne interferometry is generated using two AOMs (Isomet 1205C) that shift the laser frequency by $80\,\textrm{MHz}$ but with an offset of the heterodyne frequency of 15 kHz.

The interferometer optics on the central AEI-SAS platform are attached to a 250 mm by 250 mm Ultra-Low thermal Expansion (ULE) base plate (Ohara Clearceram-Z HS), as used in similar experiments for the LISA pathfinder mission. They are quasi-monolithically connected via a hydroxide-catalysis bond^33^. The light is brought into the vacuum vessel by polarization-maintaining optical fibers and is launched on the SPI base plate from Fibre Injector Optical Sub-assembly (FIOS)^34^. To read out the length change in the heterodyne interferometer, a phasemeter developed for the LISA Pathfinder preparatory experiments was used^35^. The signal is intrinsically calibrated to the laser wavelength. A Phasemeter Interface (PMI) converts the data to internet protocol packages that are fed into CDS. All further data processing is done using custom elements within CDS to extract and unwrap the optical phase and convert it to displacement.

#### Optical path-length difference stabilization

Two of the SPI interferometers are used as references. In Fig. 3 only one of them is shown, as the second one is redundant and it was not used during this work. These reference interferometers are confined to the quasi-monolithic assembly and detect phase noise fluctuations introduced by the input path, for example as the light propagates through the optical fibers. The signal of one is used in an Optical Path-length Difference (OPD) stabilization scheme to suppress these phase fluctuations. Figure 3 shows that a control signal is applied to a piezoelectric element driving a mirror. The light hits the mirror under normal incidence. For separation of the incoming light from the outgoing light, a polarizing beam splitter and a quarter waveplate are used. The waveplate rotates the polarization orientation from linear polarized light to circular polarization and back to an orthogonal linear polarization state, and the polarizing beam splitter spatially separates them. The mirror is attached to a long-range piezo (PI P-601), which is pre-loaded to half of its range.

#### Measurement interferometer

Each one of the two remaining interferometers measures the displacement of one of the remote AEI-SAS platforms. These signals are used for active stabilization of the relative displacement between the remote AEI-SAS and the central one. One arm remains on the base plate, while the other arm extends to the remote AEI-SAS and back. When one of the AEI-SAS moves along the beam axis the interference condition in the interferometer changes. This is detected as a phase change of the incoming heterodyne beat note compared to a digital sine wave of the same frequency and measured by the phasemeter. Continuous multi-fringe detection with a heterodyne interferometer enables tracking of motion of the AEI-SAS over lengths much greater than the laser wavelength. The interferometers have an offset in arm length of 23 m.

To reduce the laser frequency noise below the envisioned $100\,\mathrm {pm\, Hz}^{-1/2}$, an iodine-stabilized InnoLight Prometheus laser (P20NE) with integrated frequency stabilization (I2 MTS V2.0) is used. The laser frequency is stabilized to a hyperfine structure transition in molecular iodine via modulation transfer spectroscopy^36^. The projected frequency noise at $100\,\textrm{mHz}$ is $10\,\mathrm {pm\, Hz}^{-1/2}$.

Common-mode noise between the measurement and reference interferometer is present at frequencies above $1\,\textrm{Hz}$, the OPD stabilization is gain-limited due to its relatively low bandwidth. The remaining phase noise is suppressed by subtracting the reference interferometer signal from the measurement interferometer signals.

### Angular sensing

The optical levers are regarded as part of the SPI, even though they are not themselves interferometers. These optical levers are used to sense the two rotational degrees of freedom, pitch and yaw. The operating principle is simple: a laser beam is launched from one AEI-SAS and detected on the other AEI-SAS using a QPD, which measures vertical and horizontal displacements of the beam. The angular motion is amplified by the long lever arm. The translation displacement of the photodiode itself is therefore effectively suppressed.

**Figure 4:**
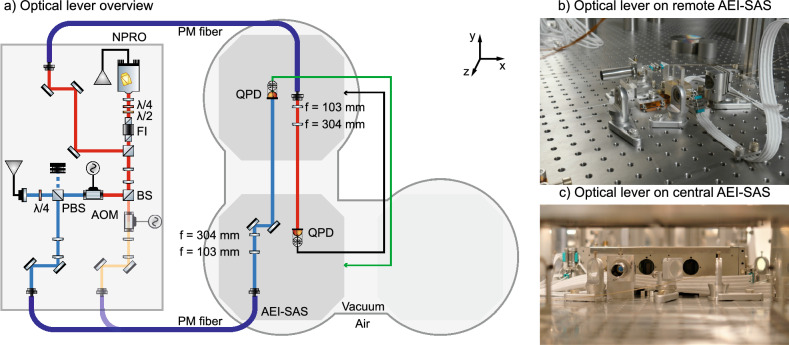
A simplified schematic of the angular sensing with the optical levers of the SPI. Each optical lever travels from one to the other AEI-SAS platforms. At the time of the experiment two AEI-SAS were installed. The feedback signals are processed in the CDS and applied to the AEI-SAS platforms for pitch and yaw control. An additional optical lever was used for out-of-loop measurements and is not shown in this figure. The photographs show parts of the optical lever hardware on the central and remote AEI-SAS.

For the central AEI-SAS platform control, laser beams from the ULE base plate are launched and directed towards the remote AEI-SAS with adjustable mirror mounts. For control of the remote AEI-SAS, dedicated fibers and fiber-optic collimators (Schäfter + Kirchhoff 60FC) were installed. Telescopes on the sending AEI-SAS platform focus the beam onto the QPD (First Sensor QP50-6-18U-TO8) on the remote AEI-SAS platform. The QPDs are mounted in linear translation mounts (Owis OH-40) for fine alignment of the QPD to the laser beam. A tube is attached in front of the QPD to reduce stray light from other experiments, see photograph in Fig. 4. The spot size on the QPDs range from 2.1 to 2.6 mm in radius, and the optical power is 1–5 mW. All photocurrents from the quadrants are individually converted to voltage signals by transimpedance amplifiers, filtered, and digitized for use in the CDS.

The individual signals from each quadrant of a QPD are combined to generate displacement signals corresponding to the pitch and yaw degrees of freedom of the AEI-SAS platforms. One optical lever therefore provides two signals. These are fed back to the AEI-SAS from which the beam was launched, see the schematic overview of Fig. 4. The optical lever signals are calibrated to match the internal sensors of the AEI-SAS and used in a feedback control system employing all-digital controllers.

### Sensitivity

**Figure 5:**
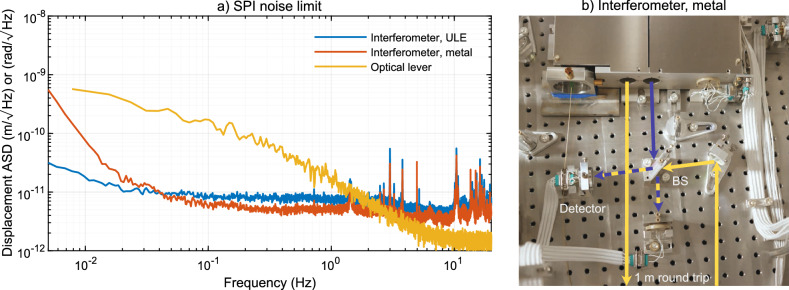
Equivalent readout noise limit of the SPI sensors for the AEI-SAS platform. The blue line shows the readout noise of the reference interferometer. The red line shows an additional interferometer, partially built using standard optical mounts, with an intentional arm length mismatch of about one meter. The stability is even better above $50\,\textrm{mHz}$ due to better fringe visibility of the interferometer. At lower frequencies, thermal drift of the mounts is probably the dominant source of noise. These measurement results show that the stability of the ULE base plate is relevant at frequencies below $50\,\textrm{mHz}$. The more interesting range for gravitational wave detectors is between $100\,\textrm{mHz}$ and $1\,\textrm{Hz}$, and here metal mounts are sufficient. The yellow line shows the readout noise limit for the optical lever in pitch and yaw. All measurements were calibrated to show the equivalent sensitivity for the AEI-SAS platform control. The metal test interferometer can be seen in the photograph.

In this section, the readout noise of the interferometers and optical levers are discussed. To characterize the readout limit of the length sensing, the length measurement of the reference interferometer is shown in Fig. 5 as the blue line. The experiment was performed with active OPD stabilization. Every interferometer has two photodetectors, one for each output port. The first photodetector of the reference interferometer is used as the sensor for the OPD stabilization. Since the bandwidth of the OPD control loop is insufficient to suppress all common-mode noise in the interferometer, it is digitally subtracted from all interferometers. The displayed readout noise is obtained with the second detector and corrected with the OPD in-loop signal. It was calibrated to match the interferometer that probes the remote AEI-SAS displacement. The equivalent readout noise for the AEI-SAS is at and below $10\,\mathrm {pm\, Hz}^{-1/2}$ for frequencies between $20\,\textrm{mHz}$ and $1\,\textrm{Hz}$.

To performance was additionally tested using an interferometer with a deliberate arm-length mismatch of about one meter that was set up on the central AEI-SAS platform. This test is sensitive to laser frequency fluctuations and it also probed the relative motion within a single AEI-SAS platform. This is important to understand whether the platform motion is a good proxy for the suspension point motion of the main optics. The thermal stability of ULE can not typically be reached in gravitational wave detectors, as most assemblies are made from aluminum and steel. The red curve shows the path-length noise of this interferometer, also calibrated to match the inter-platform displacement. Its optics were mounted in commercial aluminum mirror mounts (Radiant Dyes RD1) and a custom beam splitter holder; see the photograph in Fig. 5. It uses the same light and fiber-output collimators as the SPI. The path-length noise at frequencies above $50\,\textrm{mHz}$ was measured to be lower than for the ULE interferometer. The signal-to-noise ratio was higher due to a better mode overlap in the interferometer arms. Below $50\,\textrm{mHz}$ the noise increases, probably due to thermal drift of the metal optics mounts. The limit for the stability depends on the stiffness of the assembly, temperature drifts, and material, and it demonstrates that typical metal assemblies can be as stable as $6\,\mathrm {pm\, Hz}^{-1/2}$ above $100\,\textrm{mHz}$.

The yellow curve in the same figure shows the readout limit for the angular control of the AEI-SAS with the optical levers. The angular measurement is obtained by separately converting the photo-current from each quadrant of a single-quadrant photodiode into voltage using a four-channel transimpedance amplifier module. These signals are filtered before digitization to reduce the readout noise. Due to the limited dynamic range of the analog to digital converters, a ‘whitening’ filter with a zero at $0.33\,\textrm{Hz}$ and a pole at $33\,\textrm{Hz}$ (a lead-lag filter) is applied to the signals. It has a flat response above and below the corner frequencies. The whitening filter is inverted in the CDS after digitization. The four signals are transformed into horizontal and vertical displacement on the QPD and calibrated to the pitch and yaw degrees of freedom of an AEI-SAS using the lever arm. The equivalent readout noise range is at the nano- and picoradian level for the AEI-SAS platform. The shot noise limit is calculated to be $2\,\mathrm {prad\, Hz}^{-1/2}$.

### Suspension point motion

To verify the path-length stabilization for gravitational wave detectors, it is essential to demonstrate that the stability of the AEI-SAS platform is transferred to the suspension point of the main experiment. The signal is extracted by using the mode cleaner cavity’s feedback signal to monitor the laser frequency, which in turn follows the length of a suspended optical resonator. This combined signal path provides the independent measurement of the suspension point motion shown in the blue curve in Fig. 2.

The test was performed on an optical resonator installed in the AEI 10 m-prototype. This resonator serves as a frequency reference for the sub-SQL interferometer, which is still under construction. The resonator is composed of three mirrors. They are individually suspended as three-stage pendulums to provide a low noise length reference at Fourier frequencies above it the pendulum resonances. A photograph is shown in Fig. 2. Its length fluctuations are imposed on the laser frequency and thus distributed to the sub-SQL interferometer. A short description can be found in^29^.

The frequency reference optical resonator is an ideal test stand because it is suspended at the height of $780\,\textrm{mm}$ and is located in the corner of the platform. The AEI-SAS platform is $1.75\,\textrm{m}$ by $1.75\,\textrm{m}$ in size. The centerline of the optical resonator is about $600\,\textrm{mm}$ from the centerline of the AEI-SAS. The offset in height and position from the rotation point of the AEI-SAS makes it susceptible to the pitch and yaw of the platform. The resonator layout is schematically shown in Fig. 2. The mirror suspensions are three stages of wire suspensions, resulting in a fundamental pendulum resonance at $0.64\,\textrm{Hz}$.

The mirror suspensions have optical shadow sensors to measure the position of the suspended masses relative to their outer reference frame that is rigidly attached to the platform. As the suspension provides isolation above their natural resonances, these sensors should only be used at the pendulum resonances to dampen the motion relative to the reference frame. Below their lowest resonance frequency, the suspended masses move synchronously with the frame. The longitudinal displacement at the mirror along the beam axis is the same as at the suspension point. A measurement of the resonator length below the fundamental resonances of the suspension system is therefore a direct measure of the motion of the suspension point.

In addition, the AEI 10 m-prototype has a triangular optical resonator, the mode cleaner, that filters the laser light for the sub-SQL interferometer. Deviations of the transversal mode from the fundamental Gaussian mode, in the form of higher-order spatial modes, are suppressed by the mode cleaner^37^. Since the laser light needs to pass through this mode cleaner, it must meet the longitudinal resonance condition. The length of the mode cleaner is changed by a piezo (NAC2125-H12-C01) driven mirror in a feedback control loop. This feedback signal is used as a measure of the length change of the suspended frequency reference optical resonator. The mode cleaner consists of a Super Invar spacer with three mirrors attached to it. The mirrors form a stable optical resonator with $g=0.741$, round trip length of $530\,\textrm{mm}$, and a Finesse of about 940. It was calibrated to the SPI length readout via a driven transfer function of the AEI-SAS displacement. Without SPI stabilization, the low frequency length stability of the mode cleaner is significantly better than the suspended resonator.

## Data Availability

The datasets used and/or analyzed during the current study are available from the corresponding author on reasonable request.

## References

[1] Aasi J (2015). Advanced LIGO. Class. Quantum Gravity.

[2] Acernese F (2014). Advanced Virgo: a second-generation interferometric gravitational wave detector. Class. Quantum Gravity.

[3] Somiya K (2012). Detector configuration of KAGRA–the Japanese cryogenic gravitational-wave detector. Class. Quantum Gravity.

[4] Aso Y (2013). Interferometer design of the KAGRA gravitational wave detector. Phys. Rev. D.

[5] Abbott BP (2020). Prospects for observing and localizing gravitational-wave transients with Advanced LIGO, Advanced Virgo and KAGRA. Liv. Rev. Relat..

[6] Abbott BP (2016). Observation of gravitational Waves from a Binary Black Hole Merger. Phys. Rev. Lett..

[7] Saulson PR (1997). If light waves are stretched by gravitational waves, how can we use light as a ruler to detect gravitational waves?. Am. J. Phys..

[8] Acernese F (2010). Measurements of Superattenuator seismic isolation by Virgo interferometer. Astropart. Phys..

[9] Aston SM (2012). Update on quadruple suspension design for advanced LIGO. Class. Quantum Gravity.

[10] Matichard F (2015). Seismic isolation of advanced LIGO: review of strategy, instrumentation and performance. Class. Quantum Gravity.

[11] Wanner A (2012). Seismic attenuation system for the AEI 10 meter Prototype. Class. Quantum Gravity.

[12] Staley A (2014). Achieving resonance in the Advanced LIGO gravitational-wave interferometer. Class. Quantum Gravity.

[13] Buikema A (2020). Sensitivity and performance of the Advanced LIGO detectors in the third observing run. Phys. Rev. D.

[14] Sidles JA, Sigg D (2006). Optical torques in suspended Fabry-Perot interferometers. Phys. Lett. A.

[15] Amann F (2020). Site-selection criteria for the Einstein Telescope. Rev. Sci. Instrum..

[16] Acernese, F. *et al.* Virgo Detector Characterization and Data Quality during the O3 run. arXiv (2022). arXiv:2205.01555.

[17] Hall ED (2021). Gravitational-wave physics with Cosmic Explorer: Limits to low-frequency sensitivity. Phys. Rev. D.

[18] Punturo M (2010). The Einstein Telescope: A third-generation gravitational wave observatory. Class. Quantum Gravity.

[19] Beker MG, van den Brand JFJ, Rabeling DS (2014). Subterranean ground motion studies for the Einstein Telescope. Class. Quantum Gravity.

[20] Aso Y, Ando M, Kawabe K, Otsuka S, Tsubono K (2004). Stabilization of a Fabry-Perot interferometer using a suspension-point interferometer. Phys. Lett. A.

[21] Robertson NA, Drever RWP, Kerr I, Hough J (1982). Passive and active seismic isolation for gravitational radiation detectors and other instruments. J. Phys. E Sci. Instrum..

[22] Drever RWP, Augst SJ (2002). Extension of gravity-wave interferometer operation to low frequencies. Class. Quantum Gravity.

[23] Clark, D. E. *Control of differential motion between adjacent advanced LIGO seismic isolation platforms*. Ph.D. thesis, Stanford University (2013).

[24] Dahl K (2012). Suspension platform interferometer for the AEI 10 m prototype: concept, design and optical layout. Class. Quantum Gravity.

[25] Bergmann G (2017). Passive-performance, analysis, and upgrades of a 1-ton seismic attenuation system. Class. Quantum Gravity.

[26] Kirchhoff R (2020). Local active isolation of the AEI-SAS for the AEI 10 m prototype facility. Class. Quantum Gravity.

[27] Yu H (2018). Prospects for detecting gravitational waves at 5 Hz with ground-based detectors. Phys. Rev. Lett..

[28] Wąs M, Gouaty R, Bonnand R (2021). End benches scattered light modelling and subtraction in advanced Virgo. Class. Quantum Gravity.

[29] Goßler S (2010). The AEI 10 m prototype interferometer. Class. Quantum Gravity.

[30] Dahl K (2012). Status of the AEI 10 m prototype. Class. Quantum Gravity.

[31] Bork R (2021). advligorts: The Advanced LIGO real-time digital control and data acquisition system. SoftwareX.

[32] McNamara P, Vitale S, Danzmann K (2008). LISA Pathfinder. Class. Quantum Gravity.

[33] Elliffe EJ (2005). Hydroxide-catalysis bonding for stable optical systems for space. Class. Quantum Gravity.

[34] Bogenstahl J (2009). LTP fibre injector qualification and status. J. Phys. Conf. Ser..

[35] Heinzel G (2004). The LTP interferometer and phasemeter. Class. Quantum Gravity.

[36] Shirley JH (1982). Modulation transfer processes in optical heterodyne saturation spectroscopy. Opt. Lett..

[37] Willke B (1998). Spatial and temporal filtering of a 10-W Nd:YAG laser with a Fabry-Perot ring-cavity premode cleaner. Opt. Lett..

